# Association of BIRC5 Gene Polymorphism with the Collateral Circulation and Severity of Large Artery Atherosclerotic Stroke

**DOI:** 10.1155/2022/9177545

**Published:** 2022-01-31

**Authors:** Jianmin Huang, Xuebin Li, Jingjie Zhao, Haiyan Chen, Yanfan Yun, Guixin Yang, Yongming Jiang, Yaoxin Pan, Shengshan Yuan, Jianjun Huang, Li Su, Yingnin Wu, Dong Lu, Anding Xu, Lingzhang Meng

**Affiliations:** ^1^Stroke Center & Neurology Division, The First Affiliated Hospital of Jinan University, Guangzhou, China; ^2^Department of Neurology, The Affiliated Hospital of Youjiang Medical University for Nationalities, Baise, China; ^3^Life Science and Clinical Research Center, Affiliated Hospital of Youjiang Medical University for Nationalities, Baise City, China; ^4^Department of Radiology, The Affiliated Hospital of Youjiang Medical University for Nationalities, Baise, China; ^5^Medical Laboratory, The Affiliated Hospital of Youjiang Medical University for Nationalities, Baise, China; ^6^Center for Systemic Inflammation Research (CSIR), School of Preclinical Medicine, Youjiang Medical University for Nationalities, Baise City, China

## Abstract

**Objectives:**

The collateral circulation near the cerebral artery occlusion can contribute to the relief of the symptoms and signs of stroke. Genetic factors play a decisive role in the difference in collateral circulation. Survivin, encoded by the baculoviral inhibitor of apoptosis (IAP) repeat-containing 5 gene (BIRC5), plays an important role in maintaining long-term endothelial integrity and homeostasis and as an angiogenic factor in the treatment of vascular diseases. We hypothesized that genetic variations in the BIRC5 gene may contribute to severity by influencing the collateral circulation. This study aimed at examining how the polymorphism of the BIRC5 gene correlated with the collateral circulation and severity of large artery atherosclerotic stroke.

**Methods:**

This study enrolled 428 patients with large artery atherosclerotic stroke. There are no statistical differences in age, sex, social behavior, such as smoking and drinking, between the groups classified by the collateral circulation and by the severity of stroke (*P* > 0.01). Direct sequencing was performed for the genotyping of single nucleotide polymorphism (SNP) of BIRC5 (rs2071214). The enrolled patients were divided into several subgroups based on the collateral flow grading system from the American Society of Interventional and Therapeutic Neuroradiology/Society of Interventional Radiology (ASITN/SIR), the results of the National Institutes of Health Stroke Survey (NIHSS) (6 as a threshold), and the score of the modified Rankin scale (mRS) (for the prediction of prognosis, 2 as a threshold). Differences among subgroups were identified through logistic regression.

**Results:**

The analysis of collateral circulation revealed the significant correlation of SNP of rs2071214 with the development of poor collateral circulation of large artery atherosclerotic stroke in the additive model (GG vs. AA, odds ratio (OR) = 3.592, 95% confidence interval (CI) = 1.410–9.150, and *P*=0.007) and the recessive model (GG vs. AA/GA, OR = 3.313, 95% CI = 1.420–7.727, and *P*=0.006). The analysis of stroke severity exposed the significant role of the SNP of rs2071214 in increasing stroke severity in the dominant model (GA/GG vs. AA, OR = 1.658, 95% CI = 1.017–2.703, and *P*=0.043) and the additive model (GA vs. AA, OR = 1.717, 95% CI = 1.021–2.888, and *P*=0.042). However, the analysis of the short-term outcome indicated that three genetic models were not associated with short-term outcomes in the additive model (GA vs. AA, *P*=0.815, GG vs. AA, and *P*=0.336), the dominant model (GA/GG vs. AA and *P*=0.589), and the recessive model (GG vs. AA/GA and *P*=0.342).

**Conclusion:**

Our findings identified the SNP of rs2071214 of the BIRC5 gene as a risk factor for the poor compensatory ability of collateral circulation and a predictor of stroke severity in large artery atherosclerotic stroke, which suggested that the SNP of rs2071214 can serve as an innovative therapeutic target for patients with acute ischemic stroke.

## 1. Introduction

Stroke has caused the most deaths in China and contributed a lot to adult disability in recent years [1]. It not only reduces patients' life quality but also increases the burden on the family and society. Ischemic stroke is the main subtype of stroke with a proportion of over 80%. Its major risk factor is intracranial atherosclerotic stenosis or extracranial carotid stenosis [2]. However, no significant correlation between artery stenosis size and stroke severity in some cases of stroke has been found [3]. Our previous research concluded that the condition of collateral circulation affected the severity of clinical symptoms and signs of stroke [4]. Compared to patients with poor collateral circulation, those with good collateral circulation usually presented better results in response to therapy and stroke severity, as well as better clinical outcomes in ischemic stroke [5]. Therefore, further research of the factors that affect the collateral circulation of ischemic stroke is of great significance.

The vascular endothelial cells (VECs) play a role in regulating vascular permeability, vascular tone, and blood flow [6]. Its mechanisms include forming new blood vessels by proliferating, migrating, differentiating, and maintaining microvascular integrity and blood flow via anti-inflammatory, antithrombotic, and vasodilatory activities [7, 8]. Nonetheless, mature VECs are limited by such defects as low activity, slow growth, and renewal. Survivin, encoded by the BIRC5 gene, has the lowest molecular weight in the IAP protein family [9]. It is involved in protecting the cells by inhibiting apoptosis, regulating cell mitosis, and adapting cells to an unfavorable environment [10]. As found from a mouse model, the BIRC5 gene-transfected cells presented significant increases in the expression of cell cycle proteins, mRNA and protein expressions of survivin, cell proliferation, and invasiveness and migration activities [11]. Previous literature identifies the lack of VECs survivin as a cause of embryonic defects in angiogenesis, cardiogenesis, and neural tube closure [12]. As found from *in vivo* and *in vitro* experiments, survivin has pleiotropic effects, including enhancing cell viability and migratory capability, reducing apoptosis, and stimulating angiogenesis [13]. Moreover, the total inactivation of the BIRC5 gene was found to result in early embryonic lethality in the mouse model [13, 14]. These studies indicate the possible correlation of BIRC5 gene mutations with the integrity and homeostasis of collateral circulation.

The correlation between the SNP of BIRC5 rs2071214 and susceptibility to ischemic stroke was reported previously [15]. The close correlation of collateral circulation with the susceptibility and severity of an ischemic stroke is worth exploring in terms of its clinical significance. Nevertheless, whether the relationship of the SNP of rs2071214 with collateral circulation and severity is effective among ischemic stroke patients remains unclear. Herein, the potential association of the allelic variation of BIRC5 rs2071214 with the collateral circulation and severity of patients with ischemic stroke was explored deeply to examine the possible mechanism.

## 2. Materials and Methods

### 2.1. Study Subjects

Herein, 428 patients of acute ischemic stroke, who had disease onset from January 2019 to May 2021, were collected from the Neurology Department of the affiliated Hospital of Youjiang Medical University for Nationalities. The patients enrolled met the following criteria: (1) meeting the World Health Organization (WHO) diagnostic standard of acute ischemic stroke; (2) suffering from the first-onset cerebral infarction within 7 days; (3) receiving magnetic resonance imaging (MRI) scans to detect new lesions; (4) having digital subtraction angiography (DSA) examination results that showed the occlusion or severe stenosis of the common, internal, or middle cerebral artery, i.e., the responsible vessel; (5) aged between 50 years and 80 years; (6) providing consent to take part in this clinical research; (7) having the large artery-involved ischemic stroke as the cerebral infarction subtype according to the cerebral infarction classification of the Trial of Org 10172 in Acute Stroke Treatment (TOAST) [16]. No patients enrolled died in this study. Patients were excluded because of the following criteria: (1) 16 patients being diagnosed with cardiogenic cerebral embolism; (2) 21 patients suffering from intracranial hemorrhage, transient cerebral ischemia, and cerebral embolism of vasculitis; (3) 17 patients having nervous system damage that was not caused by acute cerebral infarction; (4) 26 patients having inherited metabolic disease or hereditary neurodegenerative disease; (5) 3 patients being diagnosed with serious diseases, such as a malignant tumor, abnormal liver function, severe infection, or autoimmune disorder. The whole study was summarized in the supplementary figure. This study was compiled following the relevant regulations of the Ethics Committee established by the affiliated Hospital of Youjiang Medical University for Nationalities.

### 2.2. Clinical Data Collection

The basic data of all enrolled participants were collected, including (1) demographics, such as age, sex, height, and body mass index; (2) the medical history, including hypertension history (diastolic pressure ≥90 mmHg or systolic pressure ≥140 mmHg measured more than 3 times during hospitalizatio or an existing treatment by antihypertensive drugs), diabetes mellitus history (random plasma glucose ≥11.1 mmol/l, fasting blood glucose ≥7.0 mmol/l, or the use of antidiabetic drugs for treatment), smoking/drinking history (over 10 cigarettes/50 ml of alcohol per day for five consecutive years); (3) family history of cerebrovascular disease; (4) the NIHSS score on admission (minor and moderate-to-severe stroke defined by the NIHSS score of <6 and ≥ 6, respectively); (5) the mRS score 3 months after the stroke onset (patients with mRS ≤2 were defined as favorable outcomes, and patients with mRS >2 were defined as unfavorable outcomes) [17].

### 2.3. Biochemical Examination

From the elbow vein of fasting patients in the second morning after admission, peripheral blood was collected for diagnosis. The residual blood will be processed for DNA isolation and sequencing. The related biochemical indicators were examined to obtain the concentrations of triglyceride, apolipoprotein B/A1, cholesterol, very low-/low-/high-density lipoprotein cholesterol, fasting blood glucose, and homocysteine.

### 2.4. Detection of Survivin Level

Serum was stored at −80°C till testing. The content of survivin in the sample was detected by ELISA. The recombinant survivin in the kit (purchased from Shanghai Xitang Biotechnology Co., Ltd.) was diluted (4000∼62.5 ng/L), and the standard curve was used to calculate the content of survivin in the sample. All procedures were performed strictly according to manufacturers' instructions.

### 2.5. Cerebral Collateral Circulation Was Evaluated by DSA

Within 7 days of onset, the whole of cerebral vessels was detected by DSA, and the endovascular procedure was performed by experienced interventional neurologists. Two experienced neurologists judged the results of the angiography with disagreements resolved by a third neurologist. The Warfarin-Aspirin Symptomatic Intracranial Disease (WASID) method was applied to the estimation of the intracranial arterial stenosis level [18]. The North American Symptomatic Carotid Endarterectomy Trial (NASCET) standard was applied to the estimation of the extracranial arterial stenosis level [19]. The stenosis degree ≥70% indicated severe stenosis or occlusion. The collateral flow grading system from the ASITN/SIR was adopted for assessing collateral circulation classification [20]. Patients with grades 0 to 2 and 3 to 4 were classified into two collateral circulation groups, poor, and good, respectively. The whole process was double-blinded.

### 2.6. SNP Selection and Genotyping

From the NCBI SNP database and previous studies on the relationship between the *BIRC5* gene and ischemic stroke, the SNP of rs2071214 was selected. Following the operation manual, we extracted the total DNA from the leukocytes from the peripheral venous blood by the Tiangen DNA kit (Tiangen Biotech, Beijing, China). The target DNA fragment was amplified using the PCR technique. Electrophoresis was conducted to confirm PCR products. Genotyping was completed by direct sequencing of Shanghai Tianhao biotechnology company (Shanghai, China). For quality control, a random 5% of the sample was selected for repeat genotyping, which presented completely consistent results.

### 2.7. Statistical Analysis

We used the SPSS 21.0 software (SPSS Inc., Chicago, Illinois, USA) for statistical analyses. Medians (quartiles) and mean ± standard deviation (SD) were used to express continuous variables. Two-group comparisons were performed by Mann–Whitney test or student's *t*-test after Gaussian distribution testing. The percentage (%) was used as an expression of categorical variables. A proportion comparison was performed using the Chi-square test. With the use of OR and 95% CI, we performed multivariate logistic regression analysis and estimated the association of genotypes with AIS collateral circulation, severity, and short-term outcome, and the relevant indicators were adjusted. One-way ANOVA was used to analyze the differences among three groups, followed by the LSD test. Statistical significance was embodied in the *P*-value <0.05.

## 3. Results

### 3.1. Distribution of the Intracranial and Extracranial Vascular Stenosis

DSA examination confirmed the degree of vascular stenosis at ≥70% among all patients. The proportions of MCA stenosis (41.76%) were the most frequent type, and the CCA stenosis (6.50%) was the least one. EC-ICA stenosis and IC-ICA stenosis occupied about 30% and 20%, respectively. Details are in Table 1.

### 3.2. Correlation between Collateral Circulation and Stenosis

Here, 428 patients were divided by the result of DSA examination into two collateral circulation groups, namely poor (250, 58.41%) and good (178, 41.59%), according to the ASITN/SIR collateral flow grading system. A larger proportion of patients with occluded blood vessels were in the good group (162, 91.01%), compared with the poor group (38,15.20%). In addition, in contrast to the poor group (212, 84.80%), the good group has much fewer patients with 70% ≤Stenosis <100% (16, 8.99%). The proportion of vascular stenosis was significantly different among the good and poor collateral circulation groups (*χ*^2^ = 240.052, *P* ≤ 0.001). The specific information is shown in Table 2.

### 3.3. Comparison of Survivin Level

**Figure 1:**
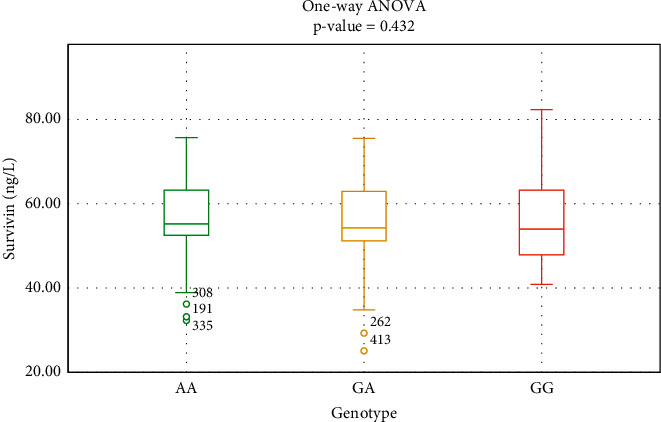
Survivin level in relation to the three genotypes of rs2071214.

According to the BIRC5 SNP of rs2071214 sequencing results, 428 patients were divided into an AA genotype group (155, 36.21%), a GA genotype group (200, 46.73%), and a GG genotype group (73, 17.06%). There was no significant difference in survivin levels among the AA group (57.06 ± 8.61), the GA group (55.81 ± 9.12), and the GG group (56.23 ± 9.58) (*F* = 0.842, *P*=0.423), as shown in Figure 1.

### 3.4. Association between BIRC5 SNP of rs2071214 and the Capacity of Collateral Circulation

The basic information of patients in the two groups is shown in Table 3. Compared with the good collateral circulation group, the poor group had a lower proportion of patients with hypertension (64.40% vs. 76.40%, *P*=0.008), and a higher HDL-C (1.11 ± 0.29 vs. 0.99 ± 0.31, *P* ≤ 0.001).  Table 4 illustrates the association between the BIRC5 SNP of rs2071214 and the capacity of collateral circulation. As found by multivariate logistic regression analysis after adjustment for age, sex, hypertension, HDL-C, and vascular stenosis degree, the GG genotype of rs2071214 significantly increased the risk of the poor group (additive model: GG vs. AA, OR = 3.592, 95% CI = 1.410–9.150, and *P*=0.007; recessive model: GG vs. AA/GA, OR = 3.313, 95% CI = 1.420–7.727, and *P*=0.006) (Table 4).

### 3.5. Association between BIRC5 SNP of rs2071214 and Stroke Severity

The baseline characteristics of the two groups of patients classified by NIHSS scores are shown in Table 5. Compared with the patients with NIHSS ≥6, the patients of NIHSS <6 had a younger age (62.56 ± 10.64 vs. 64.77 ± 10.48, and *P*=0.032), a higher HDL-C (1.11 ± 0.35 vs. 1.03 ± 0.27, and *P*=0.012), a higher proportion of patients with good collateral circulation (68.23% vs.19.92% and *P* ≤ 0.001), and more patients with vascular occlusion (61.46% vs.34.75% and *P* ≤ 0.001). The association between the BIRC5 SNP of rs2071214 and stroke severity is shown in Table 6. From the multivariate logistic regression analysis after the adjustment for age, smoking, HDL-C, collateral circulation, and vascular stenosis degree, we found the correlation of rs2071214 mutation with a higher risk of stroke severity (additive model: GA vs. AA, OR = 1.717, 95% CI = 1.021–2.888, and *P*=0.042; dominant model: GA/GG vs. AA, OR = 1.658, 95% CI = 1.017–2.703, and *P*=0.043) (Table 6).

### 3.6. Association between BIRC5 SNP of rs2071214 and Short-Term Outcome

The baseline characteristics of 428 patients grouped by mRS are shown in Table 7. Of these patients, 176 patients (41.12%) achieved a favorable outcome (mRS of 0–2). Compared with the patients with a favorable outcome, the patients with an unfavorable outcome (mRS of 3–6) had a higher HDL-C (1.09 ± 0.32 vs. 1.03 ± 0.29, and *P*=0.049), a higher LDL-C (2.93 ± 0.98 vs. 2.65 ± 0.98, and *P*=0.004), and a higher proportion of patients with severe stroke (NIHSS ≥ 6) (68.65% vs.38.80%, and *P* ≤ 0.001). The genotypes of BIRC5 SNP of rs2071214 and their association with the short-term outcome are shown in Table 8. From the multivariate logistic regression analysis after the adjustment for HDL-C, LDL-C, and NIHSS scores, we found three genotypes were not associated with the short-term outcome (additive model: GA vs. AA, *P*=0.815, GG vs. AA, and *P*=0.336; dominant model: GA/GG vs. AA and *P*=0.589; recessive model: GG vs. AA/GA and *P*=0.342) (Table 8).

## 4. Discussion

The collateral circulation near the cerebral artery occlusion plays a major role in reducing ischemia in the penumbra area [21]. The blood flow redistribution capacity of the collateral circulation is determined by the number and diameter of native collaterals [22]. Recent studies have confirmed the determinant role of genetic factors in the difference of native collateral circulation among mice samples [23]. Herein, a significantly higher percentage of patients with GG genotype was found from the poor collateral circulation group than in the good group. It reveals that for the poor compensatory collateral circulation, the BIRC5 SNP of rs2071214 is a risk factor. Moreover, compared with the patients with NIHSS ≥ 6, the patients with a GG genotype had a significantly higher percentage, suggesting that the BIRC5 GG genotype of rs2071214 was negatively correlated with stroke severity. In other words, it could predict the stroke severity of patients with acute ischemic stroke.

VECs regulate leukocyte trafficking, blood coagulation-fibrinolytic system, and vascular tone, thereby preventing vascular disorders and maintaining vascular homeostasis [24]. VEC damage and inflammation are more likely to increase vascular permeability and disrupt vascular integrity. The development of collateral circulation is found by previous literature to correlate with the complex physiological process of VECs. Nonetheless, mature VECs are subject to the defects of low activity, slow growth, and slow renewal under pathological conditions. A key factor in angiogenesis is the persistence of VECs viability, a complex pathophysiological process covering specialized antiapoptotic genes' expressions and cell mitosis regulation [25]. Cell mitosis is a complex cell cycle, transiting cell cycle-related proteins from the G0/G1, S phase to the G2-M phase. Its regulation is achieved by a complex network of cyclins and cyclin-dependent kinases (CDKs), promoting cell cycle progression by formation and activation [26]. Apoptosis means cells' spontaneous and orderly death under the control of genes, which was induced by the activation of cysteine proteases. Studies have demonstrated the necessity of preventing VECs apoptosis and promoting VECs migration and invasion for angiogenesis [27]. Therefore, how to endow VECs with antiapoptosis and promote proliferation and easy migration becomes the key challenge to maintain good collateral circulation.

Recently, the importance of survivin in vascular endothelial biology has been demonstrated. During the metaphase and anaphase of mitosis, survivin regulates the aggregation and movement of spindle microtubules and cytokinesis [28, 29]. Some studies confirmed the participation of survivin in the progression of the cell cycle from the finding that the expression of survivin increased in the G1 phase and reached the peak in the G2/M phase [30]. In the BIRC5 gene-transfected cells, the significantly increased expressions of survivin at mRNA and protein levels, significantly reduced apoptotic cells, downregulated expressions of cleaved-caspases 8, 3, and 9, and the cell viability and proliferation promoted by survivin overexpression were identified [11]. In addition, the increased migrating and invasive cells and the upregulated expressions of MMP-2, -7, and -9 in the BIRC5 gene-transfected cells at a significant level suggested that the BIRC5 gene transfection promoted the invasion of VECs by increasing MMP expression [31]. By rat experiments, after cerebral ischemia, the higher expressions of survivin protein in microvasculature were observed, revealing the protein's important biological and therapeutic significance in cerebral ischemia [14]. Additionally, the possible roles of survivin in maintaining long-term endothelial integrity and homeostasis and as an angiogenic factor for therapeutic angiogenesis of vascular diseases were revealed by previous experiments.

Genetic studies have confirmed that gene expression is affected by gene polymorphism within the promoter and/or the gene's other regulatory regions. It means, the polymorphisms of the BIRC5 gene have a possible influence on the structure and function of survivin, thus regulating the occurrence and development of various diseases by changing the survivin activity. Moreover, rs2071214 located in the exon 4 of the BIRC5 gene is identified as a risk factor of tumor pathogenesis [29]. The association between the BIRC5 gene polymorphism and diseases has been repeatedly reported. Previous studies have found that rs2071214 mutation could lead to the change of amino acid from Lys to Glu at codon 129 of exon 4. Homodimerization is induced by the acetylation of lysine 129 position of survivin, while deacetylation promotes the formation of survivin monomers heterodimerizing with CRM1. Moreover, it also facilitates nuclear export [32]. As a gene target of STAT3, survivin is of significance to the neuroprotection of estradiol, as evidenced by the finding that the shRNA of survivin reversed the neuroprotection in the rat models of cerebral ischemia [33]. The mutation of rs2071214 in the neuroblastoma cell lines was found to downregulate survivin synthesis in the nucleus [34]. For patients suffering from non-small cell lung cancer, rs3764383 polymorphism has been proved to be a negative factor of poor prognosis [35]. It is effective to help patients with ischemic stroke lower the risk of hemorrhagic transformation [36]. Survivin was upregulated when angiogenic growth factors, such as VEGF and angiopoietin-1 were released in the vascular endothelial cells [14]. Whether the biologic role of amino acid alterations correlated with BIRC5 polymorphism in collateral circulation remains unclear. The functional consequences of the BIRC5 mutation may play a critical role in collateral circulation integrity and homeostasis by inhibiting VEC apoptosis, altering enzymatic activity, and modifying the characteristics of Ang-1, VEGF, and other angiogenic growth factors. The identification of the factors that promote stroke will help prevent and control this disease, [37] while this study provides the clinical evidence that the polymorphism of BIRC5 could potentially serve as a biomarker to evaluate the severity of stroke.

### 4.1. Limitations

The limitations of the present study are listed below. Firstly, the research objects were the small case number and the inherent defects of its retrospective study nature. Therefore, a larger sample size is needed for further studies. Secondly, only one site of rs2071214 was considered, failing to completely represent the polymorphism of other loci. Moreover, the mechanism of rs2071214 polymorphism affecting collateral circulation was not investigated.

## 5. Conclusions

In conclusion, our results confirm that BIRC5 polymorphism is associated with the collateral circulation and severity of large artery atherosclerotic stroke. Furthermore, patients with rs2071214 variant genotype presented poor collateral circulation and stroke severity. Further exploration is still needed to examine the mechanism of the SNP of rs2071214 on collateral circulation.

## Figures and Tables

**Table 1:** The distribution of vascular stenosis type in all subjects.

Group	Stenosis (cases, %)
	692

CCA	45 (6.50%)
EC-ICA	206 (29.77%)
IC-ICA	152 (21.97%)
MCA	289 (41.76%)

CCA, common carotid artery; EC-ICA, extracranial internal carotid artery; IC-ICA, intracranial internal carotid artery; MCA, middle cerebral artery.

**Table 2:** Correlation between the collateral circulation condition and the vascular stenosis degree.

Stenosis degree	Good group (cases, %)	Poor group (cases, %)

70% ≤ Stenosis < 100%	16 (8.99%)	212 (84.80%)
Occlusion	162 (91.01%)	38 (15.20%)
Total	178	250
*χ* ^2^	240.052	
*P*	≤0.001	

**Table 3:** Baseline characteristics of patients grouped by the capacity of collateral circulation.

Variables	Poor group (0–2) *n* = 250	Good group (3–4) *n* = 178	*P*

Age, year (mean ± SD)	64.53 ± 10.41	62.69 ± 10.76	0.076
Sex (male) (%)	129 (51.60)	107 (60.10)	0.081
Smoking (%)	85 (34.00)	67 (37.60)	0.438
Drinking (%)	94 (37.60)	62 (34.80)	0.558
Diabetes (%)	53 (21.20)	38 (21.30)	0.971
Hypertension (%)	161 (64.40)	136 (76.40)	0.008
BMI ≥ 25 kg/m^2^ (%)	83 (33.20)	63 (35.40)	0.637
Total cholesterol (mmol/L) (mean ± SD)	4.35 ± 1.21	4.28 ± 1.24	0.552
Triglycerides (mmol/L) (mean ± SD)	1.59 ± 1.31	1.68 ± 1.20	0.453
HDL-C (mmol/L) (mean ± SD)	1.11 ± 0.29	0.99 ± 0.31	≤0.001
LDL-C (mmol/L) (mean ± SD)	2.88 ± 1.00	2.72 ± 0.94	0.106
Homocysteine (mmol/L) (mean ± SD)	15.74 ± 10.79	15.21 ± 6.61	0.561

SD, standard deviation; BMI, body mass index; HDL-C, high density lipoprotein cholesterol; LDL-C, low density lipoprotein cholesterol.

**Table 4:** Association of BIRC5 SNP of rs2071214 with the collateral circulation of stroke patients.

Variables	Poor group (0–2) *n* = 250	Good group (3–4) *n* = 178	*P* ^a^	OR (95% CI)^a^

rs 2071214				
*Additive model*				
AA	86 (34.40%)	69 (38.76%)	Ref	1.00
GA	110 (44.00%)	90 (50.56%)	0.685	1.150 (0.585–2.263)
GG	54 (21.60%)	19 (10.68%)	0.007	3.592 (1.410–9.150)

*Dominant model*				
AA	86 (34.40%)	69 (38.76%)	Ref	1.00
GA/GG	164 (65.60%)	109 (61.24%)	0.194	1.526 (0.807–2.886)

*Recessive model*				
AA/GA	196 (78.40%)	159 (89.32%)	Ref	1.00
GG	54 (21.60%)	19 (10.68%)	0.006	3.313 (1.420–7.727)

Logistic regression analysis was used to evaluate the distribution of genotype frequencies of rs2071214 polymorphisms in good collateral circulation and bad collateral circulation group. OR, odds ratio; CI, confidence interval; Ref, reference; SNP, single nucleotide polymorphism.^a^ Adjusted for age, sex, hypertension, HDL-C, vascular stenosis degree.

**Table 5:** Baseline characteristics of patients grouped by NIHSS scores.

Variables	NIHSS < 6 *n* = 192	NIHSS ≥ 6 *n* = 236	*P*

Age, year (mean ± SD)	62.56 ± 10.64	64.77 ± 10.48	0.032
Sex (male) (%)	111 (57.81)	125 (52.97)	0.316
Smoking (%)	60 (31.25)	92 (38.99)	0.096
Drinking (%)	64 (33.33)	92 (38.99)	0.227
Diabetes (%)	37 (19.27)	54 (22.88)	0.364
Hypertension (%)	126 (65.62)	171 (72.46)	0.127
BMI ≥ 25 kg/m^2^ (%)	68 (35.42)	78 (33.05)	0.608
Total cholesterol (mmol/L) (mean ± SD)	4.34 ± 1.14	4.30 ± 1.29	0.789
Triglycerides (mmol/L) (mean ± SD)	1.65 ± 1.17	1.62 ± 1.34	0.808
HDL-C (mmol/L) (mean ± SD)	1.11 ± 0.35	1.03 ± 0.27	0.012
LDL-C (mmol/L) (mean ± SD)	2.78 ± 0.99	2.84 ± 0.99	0.535
Homocysteine (mmol/L) (mean ± SD)	14.72 ± 8.47	16.16 ± 9.86	0.079
Collateral circulation (good) (%)	131 (68.23)	47 (19.92)	≤0.001
Vascular stenosis (occlusion) (%)	118 (61.46)	82 (34.75)	≤0.001

NIHSS, National Institutes of Health Stroke Survey; SD, standard deviation; BMI, body mass index; HDL-C, high density lipoprotein cholesterol; LDL-C, low density lipoprotein cholesterol.

**Table 6:** Association of BIRC5 SNP of rs2071214 with stroke severity.

Variables	NIHSS < 6n = 192	NIHSS ≥ 6 *n* = 236	*P* ^a^	OR (95% CI)^a^

rs 2071214				
*Additive model*				
AA	81 (42.19%)	74 (31.35%)	Ref	1.00
GA	85 (44.27%)	115 (48.73%)	0.042	1.717 (1.021–2.888)
GG	26 (13.54%)	47 (19.92%)	0.263	1.496 (0.739–3.027)

*Dominant model*				
AA	81 (42.19%)	74 (31.36%)	Ref	1.00
GA/GG	111 (57.81%)	162 (68.64%)	0.043	1.658 (1.017–2.703)

*Recessive model*				
AA/GA	166 (86.46%)	189 (80.08%)	Ref	1.00
GG	26 (13.54%)	47 (19.92%)	0.768	1.101 (0.580–2.092)

Logistic regression analysis was used to evaluate the distribution of genotype frequencies of rs2071214 polymorphisms in NIHSS < 6 and NIHSS ≥ 6 group. OR, odds ratio; CI, confidence interval; Ref, reference; NIHSS, National Institutes of Health Stroke Survey.^a^ Adjusted for age, smoking, HDL-C, collateral circulation, vascular stenosis degree.

**Table 7:** Baseline characteristics of patients grouped by short-term outcome.

Variables	mRS ≤ 2 *n* = 176	mRS > 2 *n* = 252	*P*

Age, year (mean ± SD)	64.22 ± 10.26	63.45 ± 10.82	0.388
Sex (male) (%)	88 (50.00)	148 (58.73)	0.074
Smoking (%)	63 (35.80)	89 (35.32)	0.919
Drinking (%)	63 (35.80)	93 (36.68)	0.814
Diabetes (%)	42 (23.86)	49 (19.44)	0.272
Hypertension (%)	121 (68.75)	176 (69.84)	0.810
BMI ≥ 25 kg/m2 (%)	59 (33.52)	87 (34.52)	0.830
Total cholesterol (mmol/L) (mean ± SD)	4.23 ± 1.17	4.39 ± 1.26	0.198
Triglycerides (mmol/L) (mean ± SD)	1.63 ± 1.25	1.64 ± 1.28	0.948
HDL-C (mmol/L) (mean ± SD)	1.03 ± 0.29	1.09 ± 0.32	0.049
LDL-C (mmol/L) (mean ± SD)	2.65 ± 0.98	2.93 ± 0.98	0.004
Homocysteine (mmol/L) (mean ± SD)	15.46 ± 9.75	15.56 ± 8.96	0.911
NIHSS ≥ 6	63 (38.80)	173 (68.65)	≤0.001

mRS, modified Rankin Scale; SD, standard deviation; BMI, body mass index; HDL-C, high density lipoprotein cholesterol; LDL-C, low density lipoprotein cholesterol; NIHSS, National Institutes of Health Stroke Survey.

**Table 8:** Association of BIRC5 SNP of rs2071214 with short-term outcome.

Variables	mRS ≤ 2 *n* = 176	mRS > 2 *n* = 252	*P* ^a^	OR (95% CI)^a^

rs 2071214				
*Additive model*				
AA	71 (40.34%)	84 (33.33%)		1.00
GA	81 (46.02%)	119 (47.22%)	0.815	1.056 (0.668–1.670)
GG	24 (13.64%)	49 (19.44%)	0.336	1.356 (0.729–2.521)

*Dominant model*				
AA	71 (40.34%)	84 (33.33%)		1.00
GA/GG	105 (59.66%)	168 (66.67%)	0.589	1.126 (0.731–1.736)

*Recessive model*				
AA/GA	152 (86.36%)	203 (80.56%)		1.00
GG	24 (13.64%)	49 (19.44%)	0.342	1.314 (0.748–2.307)

Logistic regression analysis was used to evaluate the distribution of the genotype frequencies of rs2071214 polymorphisms in mRS ≤ 2 and mRS > 2 group. OR, odds ratio; CI, confidence interval; Ref, reference; mRS, modified Rankin Scale. ^a^Adjusted for HDL-C, LDL-C, and NIHSS scores.

## Data Availability

The datasets and code generated or analyzed in this study are available from the corresponding author upon reasonable request.

## Supplementary Materials

Supplementary Materials
*Supplementary Figure*. Flow chart of the study design.
