# Supplementary figures and images for: Association of BIRC5 Gene Polymorphism with the Collateral Circulation and Severity of Large Artery Atherosclerotic Stroke
Source: Int J Clin Pract. 2022 Jan 31;2022:9177545. doi: 10.1155/2022/9177545 (PMC9159164; doi:10.1155/2022/9177545)

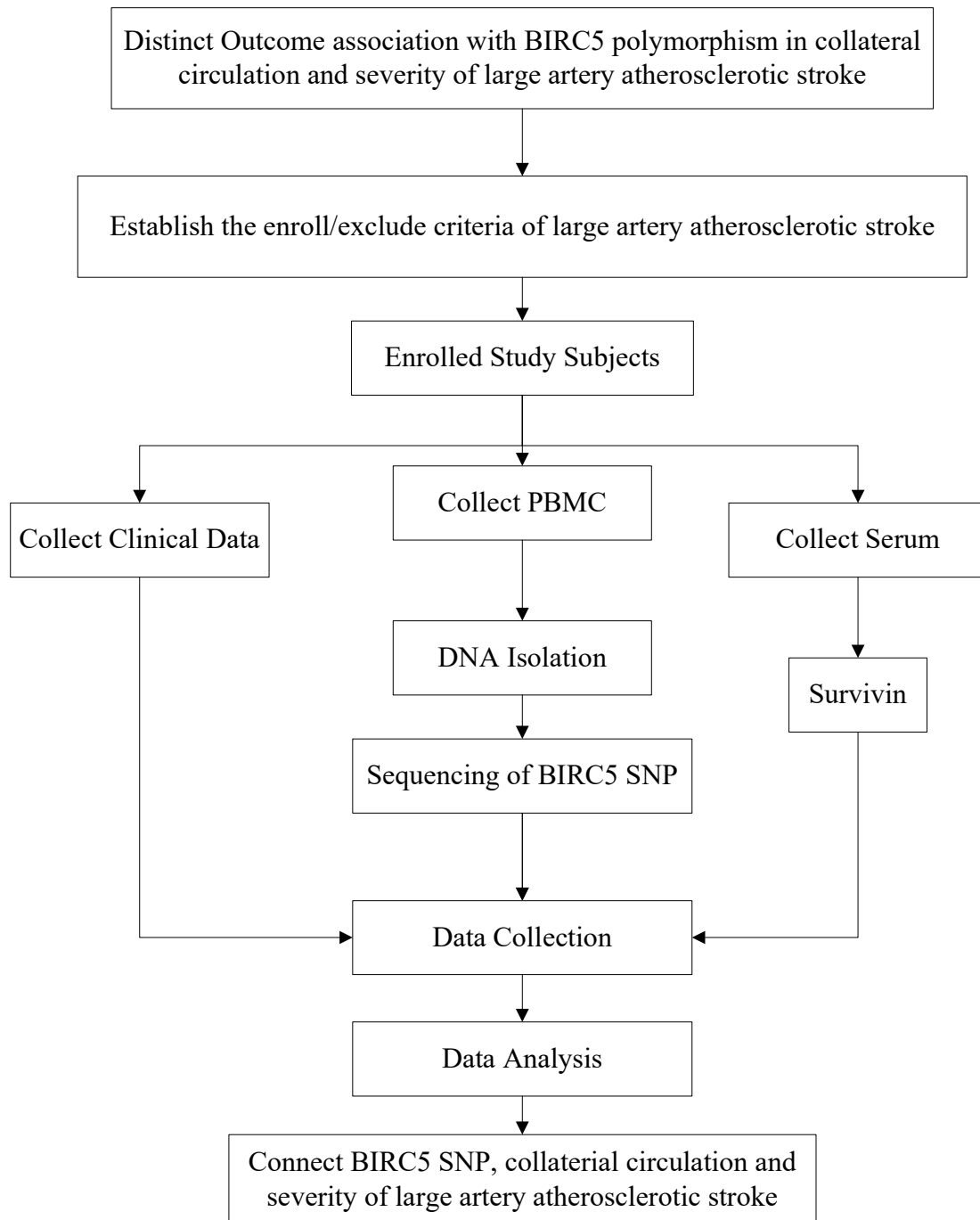

Supplement: Supplementary Materials — Supplementary Figure. Flow chart of the study design. [file 9177545.f1.pdf]
